# The Doctor's Story

**Published:** 1886-06

**Authors:** 


### THE DOCTOR'S STORY.

Mrs. Rogers lay in her bed,
Bandaged and blistered from foot to head,
Bandaged and blistered from head to toe,
Mrs. Rogers was very low.
Bottle and saucer, spoon and cup,
On the table stood bravely up;
Physic of high and low degree;
Calomel, catnip, boneset tea—
Everything a body could bear,
Excepting light and water and air.

I opened the blinds; the day was bright,
And God gave Mrs. Rogers some light,
I opened the window; the day was fair,
And God gave Mrs. Rogers some air.
Bottles and blisters, powders and pills,
Catnip, boneset, syrup and squills,
Drugs and medicines, high and low,
I threw them as far as I could throw.
"What are you doing?" my patient cried;
"Frightening Death," I coolly replied.
"You are crazy!" a visitor said.
I flung a bottle at her head.

Deacon Rogers, he came to me;
"Wife is comin' round," said he,
"I really think she'll worry through;
She scolds me just as she used to do.
All the people have poohed and slurred—
And the neighbors have had their word;
'Twas better to perish, some of 'em say,
Than be cured in such an irregular way."
"Your wife," said I, "had God's good care,
And his remedies—light and water and air,
All the doctors, beyond a doubt,
Couldn't have cured Mrs. Rogers without."

The deacon smiled and bowed his head:
"Then your bill is nothing," he said,
"God's be the glory, as you say;
God bless you, doctor, good day! good day!"

If ever I doctor that woman again,
I'll give her medicines made by men.—*Medical World.*

**The treatment** of varicose veins with Hamemelis has proved very successful for both male and female patients. Dr. B. F. Nicholls says he considers it almost a specific for varicose veins from almost any cause.

